# DNA methylation during human adipogenesis and the impact of fructose

**DOI:** 10.1186/s12263-020-00680-2

**Published:** 2020-11-26

**Authors:** Giulia Tini, Vijayalakshmi Varma, Rosario Lombardo, Greg T. Nolen, Gregory Lefebvre, Patrick Descombes, Sylviane Métairon, Corrado Priami, Jim Kaput, Marie-Pier Scott-Boyer

**Affiliations:** 1 The Microsoft Research – University of Trento Centre for Computational and Systems Biology, Piazza Manifattura 1, 38068 Rovereto, Italy; 2 Department of Mathematics, University of Trento, Via Sommarive 14, 38050 Povo, Italy; 3 Present address: Department of Experimental Oncology, IEO European Institute of Oncology IRCSS, Milan, Italy; 4 Division of Systems Biology, National Center for Toxicological Research, FDA, 3900 NCTR Road, Jefferson, AR 72079 USA; 5 Present Address: Cardiovascular Renal and Metabolism Division of MedImmune, Astrazeneca, Gaithersburg, MD 20878 USA; 6 Nestlé Institute of Health Science, Lausanne, Switzerland; 7 Department of Computer Science, University of Pisa, Pisa, Italy; 8 Present Addresses: Vydiant Inc., Folsom, CA 95630 USA; 9 Present Address: CRCHU de Québec-Université Laval, Quebec City, Québec Canada

**Keywords:** Adipocyte differentiation, DNA methylation, Differentially methylated regions, Gene expression, Integration analysis, Obesity

## Abstract

**Background:**

Increased adipogenesis and altered adipocyte function contribute to the development of obesity and associated comorbidities. Fructose modified adipocyte metabolism compared to glucose, but the regulatory mechanisms and consequences for obesity are unknown. Genome-wide methylation and global transcriptomics in SGBS pre-adipocytes exposed to 0, 2.5, 5, and 10 mM fructose, added to a 5-mM glucose-containing medium, were analyzed at 0, 24, 48, 96, 192, and 384 h following the induction of adipogenesis.

**Results:**

Time-dependent changes in DNA methylation compared to baseline (0 h) occurred during the final maturation of adipocytes, between 192 and 384 h. Larger percentages (0.1% at 192 h, 3.2% at 384 h) of differentially methylated regions (DMRs) were found in adipocytes differentiated in the glucose-containing control media compared to adipocytes differentiated in fructose-supplemented media (0.0006% for 10 mM, 0.001% for 5 mM, and 0.005% for 2.5 mM at 384 h). A total of 1437 DMRs were identified in 5237 differentially expressed genes at 384 h post-induction in glucose-containing (5 mM) control media. The majority of them inversely correlated with the gene expression, but 666 regions were positively correlated to the gene expression.

**Conclusions:**

Our studies demonstrate that DNA methylation regulates or marks the transformation of morphologically differentiating adipocytes (seen at 192 h), to the more mature and metabolically robust adipocytes (as seen at 384 h) in a genome-wide manner. Lower (2.5 mM) concentrations of fructose have the most robust effects on methylation compared to higher concentrations (5 and 10 mM), suggesting that fructose may be playing a signaling/regulatory role at lower concentrations of fructose and as a substrate at higher concentrations.

**Supplementary Information:**

The online version contains supplementary material available at 10.1186/s12263-020-00680-2.

## Background

Obesity and its comorbidities are growing worldwide epidemics [83]. A major component of modern, Westernized nutrition is the consumption of highly refined sugar [48] that has been associated to the increasing incidence of metabolic disorders [33, 45]. While overnutrition by consuming added sucrose remains the focus of more than 100 years of research, the sharp rise in obesity and diabetes since the introduction of high-fructose corn syrup (HFCS) to manufactured foods directed attention to the role of fructose in metabolism and disease development [30]. The most recent meta-analysis of 13 studies with a combined total of 49,591 participants and over 14,000 cases showed a linear association between fructose intake and metabolic syndrome (MetS). However, this adverse correlation was specific to sugar-sweetened beverages (SSB) [60] and not other fructose-containing foods (e.g., yogurt, whole fruits) indicating that the food matrix plays a significant (and expected) role in the metabolism of nutrients. Fructose metabolism and the consequent effects on the regulation of host energy balance have been extensively studied in the gastrointestinal tract, kidney, and liver [25, 64]. Human, laboratory animal, and cell culture systems have also demonstrated that fructose can be metabolized in the hypothalamus [9], innate immune system [83], cardiac and skeletal muscles ([25, 64], and adipose tissue and cells [25, 64]. Excess fructose intake leads to the development of the multiple features of metabolic syndrome [7] including fatty liver, insulin resistance, diabetes, obesity, and hypertension [25].

Adipose tissue is a key node in the physiological system of health and disease development since it stores excess energy in the form of triglyceride through (i) an increase of adipocyte size (hypertrophy) and (ii) the promotion of differentiation or adipogenesis of pre-existing adipocytes (hyperplasia) [10]. Obesity occurs as a consequence of a chronic positive energy intake which brings hypertrophy to a plateau resulting in the promotion of hyperplasia to manage with unbalance energy intakes [10]. Early research identified the peroxisomal proliferator activate receptors γ (PPARγ) and CCAAT/enhancer binding protein α (C/EBPα) as the master regulators of adipogenesis. The use of gene knockouts or knockdowns in cell culture and laboratory animals has greatly expanded the knowledge of the transcription factors, chromatin (re) modeling proteins (e.g., histones, sirtuins) and enzymes (protein acetyltransferases/deacetyltransferase and methylases/demethylases), microRNAs, and DNA methylation/demethylations that are involved in control of adipogenesis [37]. Time course experiments also demonstrated the changes in transcriptional regulation and protein-protein interaction networks that occur during differentiation from pre-adipocytes to adipocytes [51].

The role of DNA methylation has been the main focus of the control of gene expression because it had previously been thought to be a “stable” epigenetic mark. DNA methyltransferases (DNMTs) transfer the methyl group from S-adenosylmethionine to the 5 position of the cytosine primarily in CpG dinucleotides forming 5-methylcytosine (5mC) [31]. Methylation in CpG-enriched regions, called CpG islands, provides regulatory mechanisms of gene expression and is essential for cell differentiation and tissue integrity [3]. The effect of methylation on gene expression depends on where methylation occurs: high methylation levels in promoters normally repress gene transcription [38], while methylation within intronic and exonic regions of a gene body is positively correlated with expression [32]. DNA methylation can be affected by environmental factors such as lifestyle and diet, particularly by choline, betaine, folate, riboflavin, and vitamin B12 which are metabolized in the one-carbon cycle that produces S-adenosylmethionine, the primary methyl donor [8]. The relatively recent discovery that 5mC can be oxidized to 5-hydroxymethylcytosine (by ten-eleven translocation (TET) methylcytosine dioxygenase) as the first step in functional demethylation has revealed a more dynamic nature of DNA methylation [76].

Altered DNA methylation has been associated with chronic diseases linked to unbalanced diets such as obesity [3, 71], increased body mass index (BMI) [15], and hepatic steatosis [12]. In addition, a relationship between the methylation of genes involved in the circadian clock system and obesity, metabolic syndrome, and weight loss has been described [44].

The controversial link between increased consumption of fructose in human diets and the obesity epidemic [5] stimulated research that tested the detrimental impact of this carbohydrate on insulin resistance and adipocyte differentiation, two key processes to maintain metabolic health [36, 39]. The role of DNA methylation in fructose-induced metabolic syndrome and DNA methylation status in response to fructose has not been well characterized. Increased consumption of fructose has been shown to induce DNA methylation in *PPARα* and *CPT1A* in rat liver [53], leading to reduced expression of these genes and then to a hepatic lipid accumulation. Fructose may alter adipocyte differentiation by increasing the levels of *PPARγ*, *C/EBPα*, and *FABP4*, at least in murine cells in culture [17]. While both fructose and glucose are substrates utilized to increase adiposity, fructose was shown to contribute more to weight gain in humans [63].

**Fig. 1:**
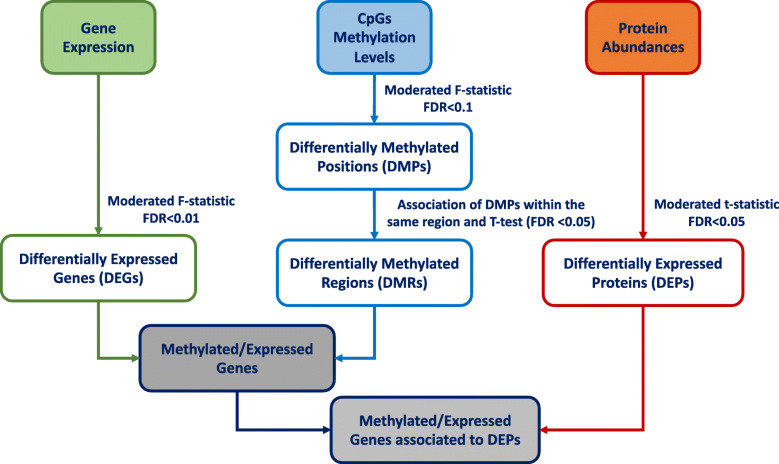
Overview of the integration analysis of the three available omics data: CpG methylation levels, gene expression, and protein abundances. The steps referring to single-omics analysis are done prior to integration and are depicted in boxes of different colors: green for gene expression, blue for methylation, and orange for proteomics. On the left of the schema, moderated *F*-statistics identify the differentially expressed genes (DEGs). The pipeline for methylation analysis is in the middle of the panel: differentially methylated positions (DMPs) were found, then DMPs in the same genomic region were assembled identifying differentially methylated regions (DMRs). The proteomics analysis (right) shows differentially expressed proteins (DEPs) identified with moderated *t*-statistics. Gray boxes at the bottom of the schema represent the steps of multi-omics integration. First, concordant changes among the epigenomic and transcriptomic levels were studied. Then, associations were made with differentially expressed proteins to find genes changing in methylation, gene expression, and protein levels

As a part of a series of studies examining the effects of fructose on adipocytes [70], we analyzed genome-wide transcriptomic and DNA methylation data at multiple time points during differentiation of the human Simpson-Golabi-Behmel syndrome (SGBS) euploid progenitor cells. Using an integrative approach (see Fig. 1), we identified genomic regions of differentially expressed genes where methylation of CpG sites differed compared to undifferentiated adipocytes.

## Results

### Genome-wide DNA methylation and transcriptomic during adipocyte differentiation

Genome-wide DNA methylation was measured using the Illumina 450K BeadChip at different time points compared to baseline during adipocyte differentiation (see Table 1 for the study design).

**Table 1:** Summary of the study design. DNA methylation and gene expression were assayed at 6 different time points for control adipocytes, proteomics only at four time points (24 and 48 h excluded). DNA methylation, gene expression, and proteins changes in the fructose-treated adipocytes were examined at 192 and 384 h, following the addition of 3 different doses of fructose (2.5 mM, 5 mM, 10 mM)

Hours	Glucose (mM)	Fructose (mM)	Methylation	Gene expression	Proteomics
0	5	0	**✓**	**✓**	**✓**
24 (1 day)	5	0	**✓**	**✓**	**━**
48 (2 days)	5	0	**✓**	**✓**	**━**
96 (4 days)	5	0	**✓**	**✓**	**✓**
192 (8 days)	5	0, 2.5, 5, 10	**✓**	**✓**	**✓**
384 (16 days)	5	0, 2.5, 5, 10	**✓**	**✓**	**✓**

The total number of genome-wide differentially methylated positions (DMPs) was identified as 8, 9, 16, 1904, and 116,637 at 24, 48, 96, 192, and 384 h, respectively, while at the same time points, the genome-wide differentially methylated regions (DMRs) were 2, 2, 4, 607, and 15,573. The corresponding differential transcriptomic analysis identified 2007, 2473, 4977, 6594, and 5237 genes at those time points (Table 2).

**Table 2:** Differentially methylated regions (DMRs) and differentially expressed genes (DEGs) at different time points across adipocyte differentiation in control adipocytes. The number of genes differentially expressed used for the integration, the number of genome-wide DMPs, and the number of CpG sites (# DMPs on DEGs) and regions (# DMRs on DEGs) with a significant change in methylation levels are displayed for each comparison. DMRs on DEGs, as well as DMPs on DEGs, are detected only at 192 and 384 h following the initiation of differentiation

Time point (h)	# DEGs	# Genome-wide DMPs	# Genome-wide DMRs	# DMPs on DEGs	# DMRs on DEGs	# DMRs not on DEGs
24	2007	8	2	–	–	2
48	2473	9	2	–	–	2
96	4977	16	4	–	–	4
192	6594	1904	607	144	57	550
384	5237	116,637	15,573	11,979	1437	14,136

**Fig. 2:**
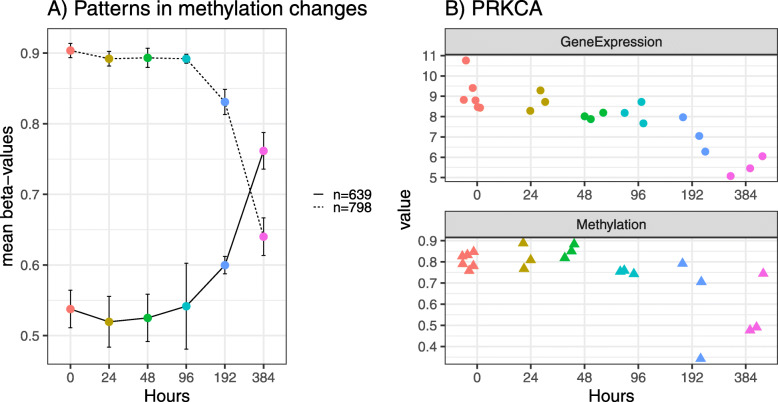
DNA methylation levels for probes in control adipocytes reveal a general decreasing level of DNA methylation for each time point. A total of 798 differentially methylated genes present the decreasing trend, but *β* values for 639 genes increase with time. Most changes occur between 192 and 384 h. **b** Change of expression (top) and methylation levels (bottom) during differentiation for the gene PRKCA. Dots represent the different replicates available for each time methylation. Different time points across differentiation are represented by different colors. **a** Methylation patterns during differentiation. Dots define averaged *β* values (absolute percentages of points)

The integration of DNA methylation and transcriptomic data identified DMPs and DMRs in genes which were differentially expressed during adipocyte differentiation. The majority of methylated positions and gene regions did not change between pre-induction (0 h) and 24, 48, and 96 h after induction (Fig. 2a). However, a large number of changes in methylation were apparent at 192 and 384 h vs baseline (Fig. 2 and Table 2).

### Methylated region location in genes

The location in the gene where DNA methylation occurs may differentially influence the gene expression [31]. At 192 h, the majority of the changes in DNA methylation occurred in the promoter region (31 out of 57 DMRs [54.4%]), while 18 genes (31.6%) were methylated in exons and 8 (14.0%) in introns (Table 3). At 384 h, 987 DMRs (68.7% of the total) were found in the promoter of the genes, 272 (18.9%) in exons, 159 (11.1%) in introns, and 19 (1.3%) in intergenic regions of genes (Table 4). The majority of the genes with differentially methylated promoter regions had decreased gene expression (e.g., up-methylation of promoter region relative to 0 h was associated with reduced gene expression), while most cases of methylation in exons or introns affected gene expression in the same direction at 384 h. Surprisingly, half of the DMRs in genes had both significant differential up-methylation and up-gene expression. This regulation could possibly be explained by other epigenetics mechanisms such as histone or other chromatin protein modifications and complex transcriptional factor regulation. Additionally, most of the DMRs on differentially expressed genes (DEGs) showing low *β* values at 384 h were found to be anti-correlated with the gene expression had (Supplementary Figure S3).

**Table 3:** Differentially methylated regions can be separated into four groups, depending on their location on the gene at 192 h. Four possible patterns can then be detected, considering gene expression (G) and methylation regulation (M), which can be increasing (↑) or decreasing (↓)

Location	M↓G↑	M↑G↓	M↑G↑	M↓G↓	Total
Promoter	15	3	6	7	**31**
Exon	10	-	4	4	**18**
Intron	3	1	1	3	**8**
Total	28	4	11	14	**57**

**Table 4:** Differentially methylated regions can be separated into four groups, depending on their location on the gene at 384 h. Four possible patterns can then be detected, considering gene expression (G) and methylation regulation (M), which can be increasing (↑) or decreasing (↓)

Location	M↓G↑	M↑G↓	M↑G↑	M↓G↓	Total
Promoter	218	368	171	230	**987**
Exon	63	48	16	145	**272**
Intron	40	25	7	87	**159**
Intergenic	8	1	3	7	**19**
Total	329	442	197	469	**1437**

### Time course pattern

Our results identified methylation patterns at different time points during the course of adipocyte differentiation. At 192 h, a total of 144 DMPs were found (see Supplementary Table S1a for the complete list) in the 6594 differentially expressed genes. Fifty-seven regions (DMRs) showed significant changes in methylation representing 0.8% of the DEGs at 192 h. (Supplementary Table S2a lists these differentially expressed genes). At 384 h, methylation changed significantly in 11,979 CpG positions (DMPs, supplementary Table S1b) and in 1437 regions (DMRs) in 1254 differentially expressed genes (23.95% of 5247 DEGs; supplementary Table S2b). A total of 130 of those genes were differentially methylated in multiple regions. Notably, 20 of the 57 DMRs at 192 h maintained their methylation status at 384 h (Figure S1 in the supplementary material) suggesting that methylation occurred before 192 h and lasted at least until 384 h. Gene enrichment analysis revealed that the top-ranked KEGG pathway for that subset of genes was glyoxylate and dicarboxylate metabolism (e.g., *SHMT1*, *GLUL*), and GO terms involved in morphogenesis, adhesion, and developmental processes. Twelve (*BCOR*, *EBF3*, *ETS2*, *GLI2*, *ITGA7*, *NPC1*, *PDXK*, *SPON2*, *GLUL* [41], *PLEKHG6*, *SHMT 1*[27], *TPD52L2* [66]) of the 20 genes have been shown to be involved in adipocyte differentiation or function, and four (*GLUL*, *PLEKHG6*, *SHMT1*, *TPD52L2*) are also involved in other types of differentiation or development processes (e.g., neurite, intestinal, cardiac, and others; see Table S3 in supplementary material).

Another group of 20 DMRs was differentially methylated/expressed only at 192 h (Figure S2 in supplementary material) and returned to the baseline level at 384 h suggesting that these were de-methylated between these time points. These genes are enriched in GO term for cell morphogenesis involved in differentiation processes (*SPINT2*, *EFNA3*, *FN1*, *MBP*) (full list of GO terms in supplementary material, Table S4). Among this group of genes, *CTDSP2* [16, 24] and *LSS* [47] have been individually studied for their roles in neuronal or sex differentiation processes, respectively. The remaining 17 DMRs were differently methylated/expressed at 192 h but were not significantly expressed or methylated at 384 h.

Genes and pathways identified in this in vitro study play roles in obesity and related conditions. Epigenome-wide association has recently shown that BMI was associated with widespread changes in DNA methylation in 210 candidate genes. Twenty-five of those candidate genes [73] were also differentially methylated at 384 h (full list in supplementary Table S5). Among these DMRs, *SREBF1*, *HOXA5*, *CPT1A*, *LPIN1*, and *PHGDH* have established roles in adipose tissue biology and insulin resistance.

### Pathway enrichment analysis

Network activity scores were calculated with NASFinder [51] for all DMRs at 384 h to investigate how pathways involved in converting a pre-adipocyte to a mature adipocyte may be influenced by methylation changes. This tool identifies statistically significant sub-networks and scores them by connecting a list of differentially expressed genes to key regulators (in this case, TFs). NASFinder analysis revealed 29 significant pathways (the 10 most significant are shown in Table 5, and all the pathways can be found in supplementary Table S6. Pathway visualizations are available on http://www.cosbi.eu/fx/2930/Visualization_C_TP384_DMR_NASFinder.zip). The pathway with the highest activity score was phospholipase C D1 in phospholipid-associated cell signaling. Several of the identified pathways are hallmarks of adipogenesis or function in adipocytes (e.g., *RXR* and *RAR* heterodimerization, *FXR* and *LCR* regulation), but others contribute new information on the differentiation process. Four of the 18 DMRs (in *ICAM1*, *PRKCA*, *RAC1*, *RAN* genes) involved in the 29 TF pathways were shown to significantly change also at the protein level. *ICAM1* maps to the integrin signaling pathway (Supplement Table S6) and may contribute to priming inflammatory processes if misregulated [43]. *PRKCA* (Fig. 2b) is involved in 11 of the 29 pathways demonstrating its central role in cell signaling. *RAC1* mapped to the semaphoring signaling (Supplement Table S6) and is a key signaling component for translocation of *GLUT4* to the cell surface [34]. *RAN* is in the non-canonical WNT signaling pathway, and this GTPase is also involved in several intracellular transport processes necessary for cell fate determination, death, proliferation, differentiation, and transformation [49]. Statistical results for the 18 DMRs involved in the pathways can be found in Table 6.

**Table 5:** The ten most significant pathways from the NASFinder TFs analysis on the 1437 significant DMRs at 384 h are displayed. The *p* value, the Network Activity Score (NAS), the identified TF, and the DMRs involved in each pathway are added

Pathways	NAS	*p* value	DMRs in the pathway	TF
BioCarta phospholipase C D1 in phospholipid-associated cell signaling	0.322	0.005	PRKCA	JUNB
BioCarta CBL-mediated ligand-induced downregulation of EGF receptors	0.191	0.008	PRKCA	MET
BioCarta activation of PKC through G protein-coupled receptor	0.186	0.003	PRKCA	NFKBIA
BioCarta apoptotic signaling in response to DNA damage	0.167	0.011	APAF1, BID, PRKCA	TP53
BioCarta role of MEF2D in T cell apoptosis	0.163	0.018	MEF2D, PRKCA	EP300
BioCarta FXR and LXR regulation of cholesterol metabolism	0.115	0.010	**ABCA1**, **NR1H3**	RXRA
BioCarta cadmium induces DNA synthesis and proliferation in macrophages	0.088	0.013	PRKCA	MYC
BioCarta TPO signaling pathway	0.064	0.029	PRKCA, **STAT5A**	STAT3
BioCarta effects of calcineurin in keratinocyte differentiation	0.060	0.022	PRKCA	SP3
Reactome regulation of gene expression by hypoxia-inducible factor	0.056	0.012	CITED2	HIF1A

Genes in bold are upregulated

**Table 6:** Statistics for the genes identified by the NASFinder as involved in the TF pathways. These genes show significant changes at 384 h in their methylation level and gene expression. Four of them are differentially expressed also at the proteomic level. Changes between 384 and 0 h are displayed for each studied omics, together with the adjusted *p* value. The region on the gene where the DMR was found is added in the table

Gene	DMR location	Delta Me	FDR Me	Log FC GE	adj Pval GE	Log FC Pr	adj Pval Pr
ABCA1	Intron	− 1.17E−01	5.39E−03	1.66E+00	1.46E−05	–	–
APAF1	Promoter	3.13E−02	1.29E−03	− 3.00E+00	7.51E−11	–	–
BID	Promoter	− 3.31E−02	4.20E−02	− 1.91E+00	7.89E−07	–	–
CITED2	Promoter	1.67E−02	1.17E−02	− 1.14E+00	3.89E−03	–	–
ETS2	Exon	− 5.49E−01	1.80E−02	− 3.29E+00	1.65E−12	–	–
HMOX1	Promoter	− 7.67E−02	4.73E−02	4.44E+00	1.23E−15	–	–
HSD17B4	Promoter	− 1.19E−01	1.31E−02	1.74E+00	7.11E−06	–	–
ICAM1	Promoter	− 4.91E−02	3.30E−03	− 2.43E+00	1.19E−08	− 7.39E−01	2.45E−05
MEF2D	Promoter	2.51E−02	1.45E−02	− 1.62E+00	1.17E−05	–	–
NCOR2*	Intron	− 1.26E−01	5.26E−03	− 1.31E+00	5.45E−04	–	–
NR1H3	Promoter	2.05E−02	2.27E−02	4.83E+00	4.54E−18	–	–
PCNA	Promoter	− 6.65E−02	4.51E−02	− 2.70E+00	2.02E−09	–	–
PRKCA	Promoter	− 7.76E−02	4.56E−02	− 3.34E+00	6.50E−13	− 1.26E+00	1.97E−06
RAC1*°	Promoter	− 9.51E−02	8.33E−03	− 3.32E+00	1.56E−12	− 8.47E−01	1.40E−05
RAN	Promoter	2.87E−02	1.25E−02	− 1.77E+00	4.33E−06	− 8.19E−01	2.36E−05
ROCK1	Promoter	− 2.05E−01	3.59E−02	− 1.16E+00	5.75E−03	–	–
SREBF1	Promoter	− 1.41E−01	3.96E−02	4.62E+00	2.43E−15	–	–
STAT5A	Promoter	− 1.30E−01	2.63E−02	3.03E+00	1.56E−11	–	–

°Protein overlapping with multiple DMRs. The most significant DMRs are displayed

*Gene overlapping with multiple DMRs. The most significant DMRs are displayed

One mechanism by which methylation can influence gene expression is by either positively or negatively altering access of transcription factor (TFs) to their binding sites [42]. Binding sites of differentially expressed transcription factors in promoters of differentially expressed genes were analyzed by data mining methods. Among the 181 transcription factors identified in the ENCODE database, 61 were found differentially expressed at 192 h and 56 at 384 h. No consistent or significant binding motifs in the DMRs in promotors at 192 h were found. However, 24 motifs were enriched in the differently methylated promoters at 384 h. Of those, nine were binding sites for differentially expressed TFs (*TFAP2A*, *ELF1*, *ETS1*, *E2F4*, *E2F1*, *NR2C2* [59], *NR2F2*, *RXRA*, *FLI1*), which are involved in a large number of intracellular processes such as E2F4’s [28] role in suppression of anti-proliferation-associated genes and E2F1-mediated [56] induction of the transcription factor PPARγ. The binding site motifs for those TFs were found in a total set of 486 DMRs (see supplementary Table S7).

Mapping these 486 DMRs to GO terms and pathways is challenging since individual genes are regulated by multiple transcription factors. However, analysis of the pattern of genes (total) methylated (up or down)/expressed (up or down) at 384 h (from supplementary Table S2b) indicated that over 50% of genes mapped to GO or pathways in metabolism categories (at *p* < 0.1). No cytoskeleton or extracellular matrix genes had the expected down/up pattern (Supplementary Table S8). More specific methylation mapping methods are needed to fully test and explain these putative associations.

### Integration of methylation, gene expression, and protein expression changes in fully differentiated adipocytes

**Fig. 3:**
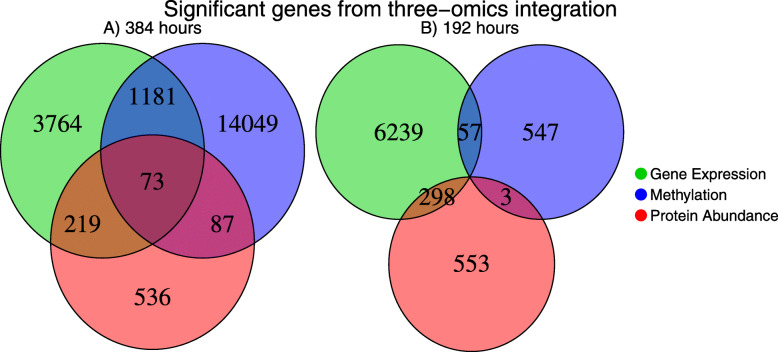
Venn diagram showing the number of genes with significant changes at **a** 384 and **b** 192 h. The green sets represent genes with a significant change in the gene expression; the blue sets correspond to the differentially methylated regions; the red diagrams represent the number of genes with significant changes at the protein abundances. The numbers in the intersections are the quantity of genes changing at two or three different omic levels. While at 384 h 73 genes changed at the transcriptomic, epigenomic, and proteomic levels, at 192 h none of the considered genes showed concordant variation among the three omics data

Proteomic data from the Somalogic platform identified and confirmed certain protein levels to the observed DNA methylation events. The results of the integration of methylation, gene expression, and protein abundances are summarized with Venn diagrams in Fig. 3. An overlap among the three omics data was found only at 384 h (Fig. 3a), while at 192 h (Fig. 3b), none of the studied genes showed consistent changes at the three different levels. A total of 73 proteins from the SomaLogic SOMAscan V1.0 panel (total of 1129 proteins) showed a significant change in the amounts between baseline and 384 h consistent with promoter, intron, and exon methylation status. Nine of these 73 proteins overlap with multiple DMRs on DEGs, resulting in a total of 84 genomic regions with proteomic, transcriptomic, and methylation changes. Eight of the 84 DMRs were upregulated at the proteomic level, while 76 are downregulated (Supplementary Table S9). Sixty-three of these were consistent with the typical pattern of methylation upregulation and downregulation of gene expression. Thirteen of the DMRs overlapping with downregulated proteins and 8 DMRs on upregulated proteins were found to have upregulated methylation and upregulation of gene expression. DNA methylation in the promoter regions typically silences genes while methylation within introns and exons is reported to positively correlate to gene expression [77]. Analysis of the pathways of the 73 proteins overlapping with DMRs will be biased since the SOMAscan version 1.0 platform is based primarily on secreted and membrane proteins (~ 66%) found in the blood with a subset derived from cellular contents (~ 33%).

### Influence of fructose on DNA methylation

The status of DNA methylation sites in SGBS adipocytes exposed to different concentrations of fructose (2.5, 5, and 10 mM) for 192 and 384 h was also assessed from the induction of differentiation (Table 7). At each fructose concentration, the methylation status of the same genes was compared to the control (without fructose at the same time point) at 192 and 384 h. Similar to the control case presented above, we performed an integration of methylation and transcriptomics. However, at 192 h, this analysis identified only 6 DMPs in the presence of 10 mM fructose (Supplementary Table S10a) and no DMRs. At 384 h, 139 and 27 DMPs were significant in response to 2.5 mM and 5 mM fructose, respectively (Supplementary Table S10b and S10c). Only 3 DMRs (*EDEM1*, *RNF145*, and *SLC3A2*) in genes differentially expressed were identified for 2.5 mM fructose, none for 5 mM fructose.

**Table 7:** Genome-wide differentially methylated regions (DMRs) found at different concentrations of fructose at 192 and 384 h

Fructose (mM)	Time point (h)	Genome-wide DMRs
2.5 vs 0	192	–
	384	26
5 vs 0	192	–
	384	7
10 vs 0	192	3
	384	–

**Fig. 4:**
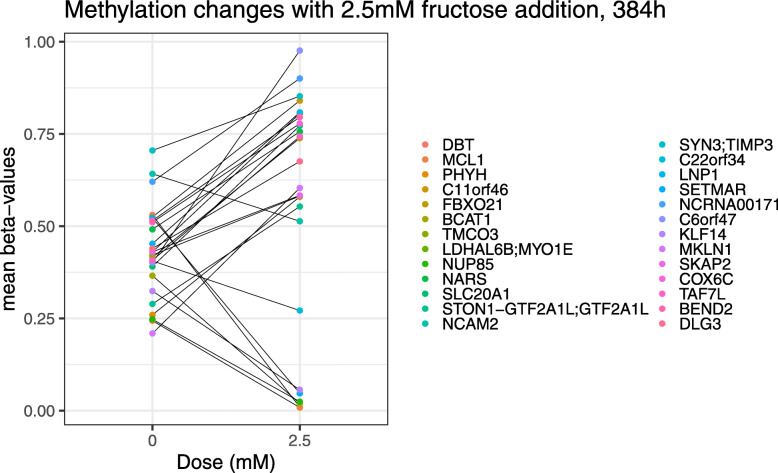
Changes in methylation levels for the 26 DMRs most affected by the addition of 2.5 mM of fructose at 384 h. Different colors represent different genes, while dots represent the mean *β* values without fructose and with 2.5 mM fructose

Since so few DMRs were found in genes differentially expressed at 192 and 384 h in response to varying fructose concentrations, genome-wide analysis of DMRs was performed to find the general effect of fructose on methylation. At 192 h, three significant genome-wide DMRs (Supplementary Table S11a) were found only for the highest dose of fructose (10 mM). At 384 h (Table 7), 26 genome-wide DMRs were detected with 2.5 mM of fructose (Fig. 4 and Supplementary Table S11b), and 7 genome-wide DMRs were detected with 5 mM of fructose (Supplementary Table S11c). Most of the DMRs identified at 2.5 mM fructose at 384 h occurred in the promoter regions of the genes (22 of the 26 DMRs). The addition of fructose resulted in the up-methylation of the majority of these gene promoters (18 of the 22 DMRs) (Table 8). Functional analysis of these 26 significant DMRs resulted in 9 enriched pathways, for example, branched-chain amino acid metabolic process and oxoacid metabolic process (Table 9).

**Table 8:** Location on the genes and regulation of the 26 DMRs found at 384 h for 2.5 mM fructose addition

Location	M↓	M↑	Total
Promoter	7	15	22
Exon	0	1	1
Intron	1	2	3
Total	**8**	**18**	**26**

**Table 9:** Enriched pathways from the DAVID analysis on the 26 DMRs found to be significant for 2.5 mM of fructose addition at 384 h. The *p* value and the genes involved in the pathways are shown in the table

Category	Term	*p* value	Genes
REACTOME_PATHWAY	R-HSA-70895: Branched-chain amino acid catabolism	0.023	*BCAT1*, *DBT*
GOTERM_BP_FAT	GO:0006367~transcription initiation from RNA polymerase II promoter	0.024	*STON1-GTF2A1L*, *GTF2A1L*, *TAF7L*
GOTERM_BP_FAT	GO:0019752~carboxylic acid metabolic process	0.025	*BCAT1*, *DBT*, *NARS*, *LDHAL6B*, *PHYH*
GOTERM_BP_FAT	GO:0043436~oxoacid metabolic process	0.026	*BCAT*, *DBT*, *NARS*, *LDHAL6B*, *PHYH*
GOTERM_BP_FAT	GO:0009083~branched-chain amino acid catabolic process	0.026	*BCAT1*, *DBT*
GOTERM_BP_FAT	GO:0009081~branched-chain amino acid metabolic process	0.030	*BCAT1*, *DBT*
GOTERM_BP_FAT	GO:0006082~organic acid metabolic process	0.035	*BCAT*, *DBT*, *NARS*, *LDHAL6B*, *PHYH*
GOTERM_BP_FAT	GO:0016054~organic acid catabolic process	0.037	*BCAT1*, *DBT*, *PHYH*
GOTERM_BP_FAT	GO:0006352~DNA-templated transcription initiation	0.039	*STON1-GTF2A1*, *GTF2A1L*, *TAF7L*

### Linked transcripts and proteins but not DMRs

Some genes (219) showed corresponding abundance changes at the protein and transcript level but not at the methylation level (Supplementary Table S12) at 384 h. DMRs at these sites were likely maintained at time points which were not analyzed in this study. Subsets of these genes mapped to 312 pathways (Benjamini adjusted *p* value < 0.05) with the secretory, membrane, and extracellular processes featuring as the most significant (< 10E−25) (see Supplementary Table S13). The GO terms may indicate a bias towards secretory proteins that constitute the SOMAscan assay panel.

### Validation with replication study

The RNA and DNA used for the transcriptomic and methylation analysis were obtained from cells plated on different plates cultured at the same time and under identical conditions. The transcriptomic and methylation analyses were generated in the same genomics facility. Since we also had access to the gene expression dataset obtained from an experiment conducted at different hours but under similar experimental conditions [51], we analyzed the integration of methylation data reported here with the gene expression dataset reported in the previous study [51]. The dataset was generated with 8 Illumina Human HT-12 version 4 BeadChips (Ilumina, Inc., San Diego, CA) hybridized with the RNA from 18 cell cultures at different time points (0, 6, 48, 96, 192, 384 h).

The secondary analysis conducted on this gene expression dataset led to similar but less significant results (Table 10). For example, significant DMRs on DEGs were found only at 384 h for a total of 198 genes over the 8279 DEGs found in the previously published study. Sixty-eight of these genes (complete list in Supplementary Table S14) were found to be differentially methylated when using data from the same experiment. Additionally, 7 of the 198 genes showed changes also at the proteomic level: three of them (THBS2, CRLF1, and C3) were found also in the analysis with the transcriptomic data used for our study.

**Table 10:** Differentially methylated positions (DMPs), differentially methylated regions (DMRs), and differentially expressed genes (DEGs) for the validation dataset. The results obtained at different time points and for different fructose doses are displayed

Time point (h)	Fructose doses (mM)	# DEGs	# DMPs on DEGs	# DMRs on DEGs
6	0	1005	–	–
48	0	5574	2	–
96	0	7334	–	–
192	0	7248	4	2
384	0	8279	452	198
192	2.5 vs 0	214	–	–
384	2.5 vs 0	2532	49	17
192	5 vs 0	79	–	–
384	5 vs 0	2466	6	3
192	10 vs 0	64	–	–
384	10 vs 0	2338	1	–

Only a few DMRs with corresponding DEGs were identified in cells exposed to fructose in this second dataset: 17 DMRs for 2.5 mM fructose and 3 for 5 mM addition at 384 h. These results are in agreement with that obtained for data from the same experiment, with DMRs only at 384 h and diminishing with fructose addition.

The two gene expression datasets were generated by separate laboratories using the same methodologies, which may explain the differences in the results [11].

## Discussion

Genome-wide changes in DNA methylation of pre-adipocytes cells induced to differentiate into mature adipocytes were analyzed to determine the role of DNA methylation in regulating gene and subsequently protein expression in control (5 mM glucose) cells. The effect of varying concentrations of fructose on methylation status and transcriptional regulation was also analyzed since fructose stimulates anabolic processes of glutamate and de novo fatty acid synthesis [70] and alters glucose metabolism to induce more energy [69].

### DMRs in control conditions

A previous report [67] concluded that DNA methylation of 84 genes was relatively stable between 0 and 240 h during human mesenchymal stem cell adipogenesis and that changes in DNA methylation were not an underlying mechanism regulating the gene expression during adipocyte differentiation. Our results largely confirm these findings since DNA methylation did not change appreciably at 24, 48, and 96 h and less than 1% at 192 h. The majority of methylation regions at 192 h were conserved at 384 h. The subset of genes with transient methylation (methylated at 192 but not at 384) occurred in genes associated to cell morphogenesis involved in the differentiation process (*SPINT2*, *EFNA3*, *FN1*, *MBP*). In addition, significant changes in methylation linked to changes in the gene expression occurred in almost 25% (1254 of 5237) of the sites analyzed at 384 h compared to pre-induction. These results are consistent with our previous metabolic [70] and transcriptomic analyses [51] that showed adipocyte-specific metabolism, and gene regulation at 192 h of differentiation becomes more “robust” by 384 h when adipocytes are in the fully differentiated state. That is, a complex set of interactions between metabolic pathways, transcriptional regulation, and DNA methylation that cause the final maturation of the adipocyte and/or DNA methylation ensures that the cell remains in its fully differentiated state (that is, it is not causal for differentiation, but rather a post-differentiation mechanism to prevent de-differentiation).

Changes in DNA methylation occurred in genes and pathways known to be involved in adipogenesis (e.g., a *PPARG* receptor linked to *RAR* and *RXR*, visualization on *http://www.cosbi.eu/fx/2930/Visualization_C_TP384_DMR_NASFinder.zip*) as well as pathways not previously associated with adipogenesis (supplement Table S4 and visualization on *http://www.cosbi.eu/fx/2930/Visualization_C_TP384_DMR_NASFinder.zip*). Upregulated genes at 384 h mapped to lipid metabolism, mitochondria, oxidoreductase, and other pathways regardless of the state of methylation. Downregulated genes mapped to cell adhesion, cell cycle, cell division, and cytoskeleton (among others) pathways and also were independent of methylation status. These results suggest that methylation is a consequence and not a driver of the final maturation stage of adipogenesis.

### Integration of DNA methylation, gene expression, and proteomics

A novel feature of this study was the analysis of DNA methylation, mRNA levels, and selected proteins at 384 h after differentiation. Seventy-three of the DMRs (out of the 84 proteins identified with Somalogic technology with the corresponding DMRs in this dataset) showed significant variation in levels indicating that DNA methylation changes were transmitted to protein levels. Of the 63 downregulated proteins and genes, DNA methylation of the majority of genes occurred in the promoter region. Methylation was also present at more than one region in some genes, for example, up-methylation of *COL18A1* occurred both in the intronic and exonic regions of the gene. Alternatively, methylation of some genes (e.g., *MRC2*, *CRLF1*, *RAC1*) occurred in the promoter or either the intronic or exonic regions. DKK [23] and other genes that have well-established roles in adipogenesis have been shown to have methylation consistent with the direction of the expression [68]. More than half of the 73 methylated genes have been identified as such in adipose tissue samples from obese and/or diabetic patients [52]. Many of these genes have not been fully characterized in adipose tissue, and a functional role in the adipogenic process remains to be defined (e.g., *CRLF1*) [82]. Methylation of several genes and their protein changes are reported here for the first time, and no literature exists describing their expression in adipose tissue (e.g., *RPS7*, *COLEC12*) or role in adipogenesis. The integration of gene methylation and their mRNA and protein levels make these likely targets and biomarkers of adipocyte differentiation and contribute to improving our understanding of this process.

### Fructose effect on methylation

Fructose has been implicated in the obesity epidemic and specifically in altering the physiology of adipocytes. Although studying the effects of nutrients in cell culture experiments is controversial because concentrations have to be estimated, the doses used in this in vitro experiment were modeled on circulating levels following fructose ingestion in humans [46, 72] and considered that local concentrations (e.g., adipose tissue associated with the intestinal tract) could be higher than reported plasma levels.

An inverse correlation was found between the number of DMRs assayed and fructose levels (Table 6). At 384 h, 26 genome-wide DMRs were detected in cells grown in 2.5 mM of fructose in the presence of 5 mM glucose. Pathway analysis mapped these genes to transcription factor processes and branched-chain amino acid (BCAA) catabolism at uncorrected *p* values of < 0.05. BCAA catabolism has a functional role in adipocyte differentiation [22], and decreased catabolism of BCAA may be related to insulin resistance, impairment of subcutaneous adipocyte hypertrophy, and associated pathologies [55, 75]. Higher circulating BCAA levels were observed in obese and diabetic patients [55].

Only 7 and 3 DMRs were found when adipocytes were exposed to 5 mM and 10 mM fructose, respectively, at 384 h post-induction of differentiation. These small numbers of genes precluded pathway analysis, but no apparent pattern was observed. Individual genes can be annotated and associated with whole body phenotypes. For example, the endoplasmic reticulum degradation-enhancing alphamannosidase-like protein1 (EDEM1) found to be differently methylated at 10 mM fructose is an endoplasmic reticulum stress (ERS) marker [58]. Acute ERS can weaken the capacity of mature adipocytes to store lipids, and chronic ERS can impair the adipogenic potential of preadipocytes [35]. Disruption of these pathways could contribute to obesity-associated morbidities such as lipid spillover, ectopic fat deposition, and ultimately insulin resistance [10]. Epigenetic modifications in ring finger protein 145 (RNF145, a metal binding proteins), also found at 10 mM fructose, were associated with BMI, waist circumference, and changes in BMI in African American adults [13].

Fructose has moderate effects on methylation at 384 h at a concentration of 2.5 mM, a 1:2 ratio with the 5 mM glucose in the culture media. However, changes in methylation decreased at equimolar doses (5 mM fructose added to basal 5 mM glucose) or 2:1 (10 mM fructose with 5 mM basal glucose). We speculate that fructose may play a signaling/regulatory role at doses less than 1:1 fructose to glucose, but at equimolar or greater levels, fructose participates as a substrate and is “shunted” to the metabolic pathways to produce stored (oleate) and released fatty acids (palmitate) as demonstrated in our previous work [70].

### Limitations

Comparing gene expression datasets generated by different laboratories is known to be problematic [11]. In this study, we compared and integrated two sets of gene expression datasets with the methylation data, of which, one of the gene expression datasets was previously published by our team while the second gene expression dataset was generated from cells cultured simultaneously with cells that were used for the methylation data. Our observation was similar to previous reports [11] in that although the trends were similar between the two separate gene expression datasets generated in different laboratories, differences were also found.

Another limitation of this study was the use of a proteomic assay technology (i.e., SomaLogic platform) that is based on a subset of proteins that are typically found in the blood. Nevertheless, these diverse high-throughput methods allow for identifying and linking changes at the chromosome level through protein levels. The integration of these diverse data types was possible by the use of a highly characterized euploid cell line [2, 19] as to more complex adipose tissue which would have multiple cell types.

#### Cell model

The cell line (SGBS) used in these experiments was derived from a male child with Simpson-Golabi-Behmel syndrome (OMIM # 312870), an overgrowth syndrome with multiple clinical features such as facial and cardiac abnormalities, macrocephaly, and organomegaly. SGBS is associated with genomic rearrangement and mutations in glypican-3, a heparan sulfate proteoglycan [65]. The cell line has retained its diploid character in culture. While cells in culture lack communication between different cells in the tissue and physiological factors from other organs that occur in vivo, SGBS cells have been used extensively as a human model for pre-adipocyte differentiation to mature adipocytes [2, 19]. Our previous study of global changes in networks and pathways during adipogenesis in SGBS corroborated the results from many studies in mouse 3T3-L1 that analyzed individual pathways or subsystems [50]. A direct comparison between SGBS and primary human white subcutaneous adipocytes from 4 obese individuals indicated quantitative but not qualitative differences in the expression of extracellular matrix proteins, some metabolic pathways, and mitochondrial respiration [81]. The differentiation process was similar between SGBS and primary adipocytes, and these direct comparisons can be informative for understanding adipogenesis in vivo.

## Conclusions

The transcriptomic and methylation data obtained in this study indicated that a majority of DNA methylation and resultant gene expression patterns are “pre-programmed” since up- or downregulation of gene expression does not appear to be causatively coupled to a promoter, exon, or intron methylation. However, promoter methylation of a subset of genes may be causatively linked to expression. In addition, the differentiation program apparently overrides the differences in the type and level of nutrients (fructose vs glucose) consistent with our previous studies of the metabolic fate of fructose [70] and the effect of fructose on glucose metabolism [69] in these cells. Further research on how nutrients affect methylation, gene expression, protein levels, and the differentiation program should focus on mesenchymal stem cells to preadipocyte transition.

## Methods

### Fructose treatment of SGBS cells

Human Simpson-Golabi-Behmel syndrome (SGBS) preadipocytes, kindly provided by Martin Wabitsch, were used and cultured as described in a previous study [70]. Briefly, the SGBS preadipocytes cells were maintained at 37 °C in a humidified incubator flushed with 5% CO_2_. The growth medium consisted of DMEM:F12 (1:1), 33 mM biotin, and 17 mM pantothenate containing 10% fetal bovine serum and 1% penicillin-streptomycin. One day post-confluence, the cells were initiated to differentiate into adipocytes by the addition of a serum-free differentiation medium containing DMEM with 25 nM dexamethasone, 500 μM IBMX, 2 μM rosiglitazone, 0.01 mg/ml human transferrin, 2× 10-8 M insulin, 10-7 M cortisol, 0.2 nM T3, 33 mM biotin, and 17 mM pantothenate. Following 4 days of maintenance in serum-free differentiation medium, the medium was changed to a serum-free adipogenic medium consisting of DMEM:F12 (1:1) with 0.01 mg/ml human transferrin, 2× 10-8 M insulin, 10-7 M cortisol, 0.2 nM T3, 33 mM biotin, and 17 mM pantothenate. Adipogenic medium is essentially similar to the differentiation medium but lacks IBMX, dexamethasone, and rosiglitazone. The medium was changed every 2 days from the initiation of differentiation. The SGBS cells differentiate into adipocytes by day 14 of differentiation as by Oil Red O staining, seen previously during the establishment of the differentiation methods of the cells, in the lab.

### Fructose treatment of SGBS cells

SGBS preadipocytes were plated at 2 × 10^5^ cells in 100-mm dishes, supplemented with 10 ml growth medium, grown to confluence, and initiated to differentiate as per Varma et al. [70]. All media used for the growth, differentiation, and maintenance of adipocytes contained a basal amount of 5 mM glucose, equivalent to the normal blood glucose concentration. Cells for RNA and DNA isolations were collected at different time points across differentiation at 24, 48, 96, 192, and 384 h. In order to determine the effects of fructose exposure on adipocytes at concentrations reported in the systemic circulation, in response to fructose-rich food [29], different doses of fructose including 2.5, 5, and 10 mM fructose were added to the media at the initiation of differentiation and maintained in the medium until the collection of cells and medium at the end points of either 192 h or until day 384 h of differentiation. The medium was changed every 2 days, from the initiation of differentiation to the collection of cell lysate experimental assays. Cell lysates from the control or fructose-treated adipocytes were collected for DNA and RNA isolations. For RNA, the media were completely aspirated from cells, and a total of 700 μl of QIAzol lysis reagent (Qiagen, Cat No./ID: 79306) was added to the cells, and the lysed cells were scraped and collected in an Eppendorf vial and sheer disrupted by passing through a tuberculin syringe about 6 times and the lysates are flash frozen. For obtaining samples for DNA isolation, the media were removed and cells were washed with PBS and aspirated to remove all PBS. The cells were gently scraped in the presence of a total of 400 μl of PBS that was added to the plates and collected using a pipette fitted with a wide mouth tip and transferred to an Eppendorf vial and flash frozen. All experiments across all our studies were conducted at the same passage numbers, and culture condition differentiations were adhered to rigorously.

### Study design

The study design is summarized in Table 1. The study was thus designed such that different concentrations of fructose were added to a medium containing 5 mM glucose to mimic the effects of different concentrations of fructose in circulation. We included a broad range of fructose concentrations (0.1–10 mM) to examine the impact of the reported concentrations of fructose in adipocytes [29, 46]. Triplicate plates of cells in culture were harvested for DNA methylation or separately for transcriptomics assays at specific time points including 24, 48, 96, 192, and 384 h for control adipocytes (six replicates were harvested at 0 h). Cultures treated with different concentrations of fructose (2.5, 5, and 10 mM) were harvested at 192 and 384 h following the initiation of differentiation (Table 1). For proteomic SOMAscan assays (version 1 [1149 somamers], Somalogic, Boulder, CO), cell lysates were obtained from cultures differentiated for 0, 96, 192, and 384 h following the induction of differentiation in a control medium supplemented with 0, 2.5, 5, and 10 mM fructose.

### DNA methylation

#### Platform

Genome-wide methylation was assessed using the Illumina Infinium HumanMethylation450K array platform (Illumina, San Diego, CA, USA) that contains a total of 485,512 CpG sites. Samples were randomly distributed over four different BeadChips to reduce batch effects. CpG sites were then annotated with the R package ilmn12.hg.19 [26] (v 0.6.0), which identified the genes and the regions on the genome. Illumina GenomeStudio software was used to extract the raw signal intensities.

#### Normalization

Preprocessing was performed with the function *preprocessIllumina* from the R package Minfi [4] 1.22.1. This method was applied to reduce Infinium I/II type bias and correct for background. Absolute percentages of methylation (*β* values) were then extracted and normalized with the SWAN method [40]. For each CpG site, averaged *β* values across the cell replicates were considered for the following analysis.

#### Differentially methylated positions

The Minfi package was used to detect differentially methylated positions. Statistical significance of CpG sites was assessed with a moderated *F*-statistic implemented in the function *dmpFinder*, which uses statistical significance cutoffs to select differentially methylated positions. Since DMPs were used as a starting point for further analysis, an FDR adjusted *p* value threshold of 0.1 was chosen.

#### Differentially methylated regions

In addition to DMPs, differentially methylated regions were identified with the R package COHCAP [74] (version 1.20.0). COHCAP functions use a list of annotated DMPs to compute the average signals from one or more CpG sites that were found in the same genomic region. A *t* test with an FDR threshold of 0.05 was applied to find those regions (DMRs) showing a significant difference in the average methylation level. The minimum number of sites needed to create a region was set at 1, while the change in methylation used to define the DMRs was left as the default values of COHCAP, which is 0.2. The possibility to use a list of annotated DMPs makes this method suitable for detecting DMRs on specific genes and thus to perform a step of integration analysis. Moreover, COHCAP uses predefined regions, allowing a more controlled analysis, in contrast to methods that define regions [57].

### Transcriptomic data

For RNA isolation, media was completely aspirated from cells and a total of 700 μl of QIAzol lysis reagent was added to the cells, and the lysed cells were scraped, collected in an Eppendorf vial, sheer disrupted by passing through a tuberculin syringe about 6 time, and the lysates flash frozen. Cells from replicate wells were used for both RNA and DNA isolation respectively.

#### Platform

The dataset was generated with 4 Illumina Human HT-12 v-4 BeadChips (Ilumina, Inc., San Diego, CA) hybridized with the RNA from 46 cell cultures at different time points (0, 24, 48, 96, 192, 384 h) and for different fructose concentration (0, 2.5 mM, 5 mM, 10 mM). RNA labeling and microarray hybridization were performed according to the manufacturer’s recommendations. Samples for RNA and DNA platform were measured on cells plated at the same time and treated similarly.

#### Normalization and filtering

The scanned data was acquired in R using the package illiminaio [61] (v 0.18.0). The non-normalized summarized bead-level data was then annotated with the R package illuminaHumanv4.db [18]. Other labeling and analysis methods were performed with the preprocessing pipeline previously described [51].

#### Differentially expressed genes

Differential expression analysis was carried out using the limma [62] R package (version 3.32.5). The probes were ranked by their log-odds scores given by empirical Bayesian moderation of sample variances with an FDR threshold of 0.01. The DEGs of fully differentiated adipocytes at 384 h in controls were further processed to identify clusters of co-expressed genes. The clusters were decomposed according to the functional categories of their genes related to the biological functions and pathways (DEG modules). The details of the procedures are described in the paper by Nassiri et al. [51].

### Proteomic data

#### Proteomic studies

SGBS preadipocytes were plated at 1 × 10^5^ cells/well in a 6-well plate and allowed to reach near confluence before adding a differentiation medium. Samples were harvested from three replicate wells at 4 different time points including day 0 just before the induction of differentiation and then days 4, 8, and 16 after the induction of differentiation.

The spent culture medium (supernatant) from the respective wells was pipetted into an Eppendorf vial, centrifuged at 13,000 RPM for 10 min at 4 °C to pellet the cell debris. The supernatant was transferred to a fresh vial and stored at − 80 °C until used. Cells were washed three times with ice-cold PBS and then lysed by the addition of 125 μl Mammalian Protein Extraction Reagent M-PER® (Pierce Biotechnology cat # 78503) supplemented with Halt™ protease inhibitors (with EDTA) (Pierce biotechnology cat # 87786) at 1× concentration and incubated for 5 min. Cell lysates were scraped and transferred to a microcentrifuge tube, centrifuged at 13,000 RPM for 10 min at 4 °C to pellet the cell debris. The clarified supernatant (lysate) obtained was transferred to a fresh tube and stored at − 80 °C. Protein concentrations in the supernatant and cell lysates were estimated using the Micro BCA Kit (Pierce Biotechnology cat # 23235) as per the recommended protocol.

#### Platform

Cell lysates in replicates were analyzed with the SOMAscan platform (SomaLogic, Inc., Boulder, CO) consisting of 1149 aptamers at different time points (0, 96, 192, and 384 h) and for different doses of fructose (0, 2.5, 5, and 10 mM). SomaLogic Inc. (Boulder, CO) performed all the proteomic assessments, and samples were analyzed as previously described [6, 20, 21, 54].

#### Differentially expressed proteins

Differentially expressed proteins were found with a robust linear model from the R package limma [62]. A threshold of 0.05 on moderated empirical Bayesian FDR was set to select significant proteins.

### Transcription factors binding sites analysis

Binding sites of transcription factors (TFs) in DMRs were identified with the function *get.enriched.motif* of the R package *ELMER* [78, 80] 1.6.0. The binding sites were searched on 181 transcription factors identified in the ENCODE database (http://amp.pharm.mssm.edu/Harmonizome/dataset/ENCODE+Transcription+Factor+Targets). The CpG sites of all DMPs were used as a background. Motifs occurring at least 10 times and with an odds ratio higher than 1 in the 95% CI were considered significant. Significant motifs from the same family were summarized with the function *motif.relevant.TFs* data from the *ELMER.data* package [79] (v 1.6.0).

### Pathway analysis

Pathways analysis was performed with NASFinder [51]. NASFinder identifies and scores statistically significant sub-networks of an interactome network connecting functionally related genes to its main regulator (e.g., receptors or transcription factors). The analysis described here was adipose-specific using transcription factors as regulators and transcripts that mapped to differentially methylated genes to find the most active pathways influenced by methylation. The *p* value threshold used to select significant pathways was < 0.05. Functional pathway enrichment analysis was also performed with DAVID [14], using default parameters and a *p* value threshold < 0.05 to analyze the fructose data.

### Integration of methylation, gene expression, and protein expression

The schema representing the different steps of the integration analysis is presented in Fig. 1.

Differentially methylated regions (DMRs), differentially expressed genes (DEGs), and significantly expressed proteins were explored at each time point and each different fructose concentration.

An integrative analysis was then performed by determining the differentially methylated regions (DMRs) that were associated with differentially expressed genes for each of the considered analysis. The location of the DMRs in the gene (promoter, exon, intron, or intergenic) was annotated with the *genomation* 1.8.0 R package [1]. Integration with protein expression was performed by determining the methylated/expressed genes that were also associated with significantly expressed proteins. All the analyses described here were performed with R version 3.4.1.

## Supplementary Information

**Additional file 1:** **Figure S1.** Methylation patterns during differentiation for the 20 genes showing significant methylation changes both at 192 and 384 hours. To better inspect the patterns, genes are separated in four panels. Dots define averaged β-values for each time-point, while different colors represent different genes. **Figure S2.** Methylation patterns during differentiation for the 20 genes significantly methylated/expressed at 192 hours (but not at 384 hours) which methylation levels return to the baseline at 384 hours. For each panel, mean β-values at each time point are represented by dots. Different colors represent different genes. **Figure S3.** Distribution of the average beta-values for the DMRs on DEGs at 384 hours. β-values of DMRs that show anti-correlated changes in methylation and gene expression are depicted in pink. The blue curve describes instead the distribution of β-values for DMRs showing the same direction of regulation in methylation and gene expression levels.

**Additional file 2.** Supplementary tables.

## Data Availability

The datasets are available on GEO (accession number GSE119539 and GSE119593). Go to https://www.ncbi.nlm.nih.gov/geo/query/acc.cgi?acc=GSE119593 Go to https://www.ncbi.nlm.nih.gov/geo/query/acc.cgi?acc=GSE119539
