# Supplementary material for: DNA methylation during human adipogenesis and the impact of fructose
Source: Genes Nutr. 2020 Nov 26;15:21. doi: 10.1186/s12263-020-00680-2 (PMC7691080; doi:10.1186/s12263-020-00680-2)
Supplement: Supplementary file 1 — Additional file 1: Figure S1. Methylation patterns during differentiation for the 20 genes showing significant methylation changes both at 192 and 384 hours. To better inspect the patterns, genes are separated in four panels. Dots define averaged β-values for each time-point, while different colors represent different genes. Figure S2. Methylation patterns during differentiation for the 20 genes significantly methylated/expressed at 192 hours (but not at 384 hours) which methylation levels return to the baseline at 384 hours. For each panel, mean β-values at each time point are represented by dots. Different colors represent different genes. Figure S3. Distribution of the average beta-values for the DMRs on DEGs at 384 hours. β-values of DMRs that show anti-correlated changes in methylation and gene expression are depicted in pink. The blue curve describes instead the distribution of β-values for DMRs showing the same direction of regulation in methylation and gene expression levels. [file 12263_2020_680_MOESM1_ESM.docx]

**Supplementary material**

**DNA methylation during human adipogenesis and the impact of fructose**

Giulia Tini^1,2^, Vijayalakshmi Varma^3^, Rosario Lombardo^1^, Greg T. Nolen^3^ ,Gregory Lefebvre^4^, Patrick Descombes^4^, Sylviane Métairon^4^, Corrado Priami^1,5^, Jim Kaput^4^, Marie-Pier Scott-Boyer^1^

**Supplementary figures**


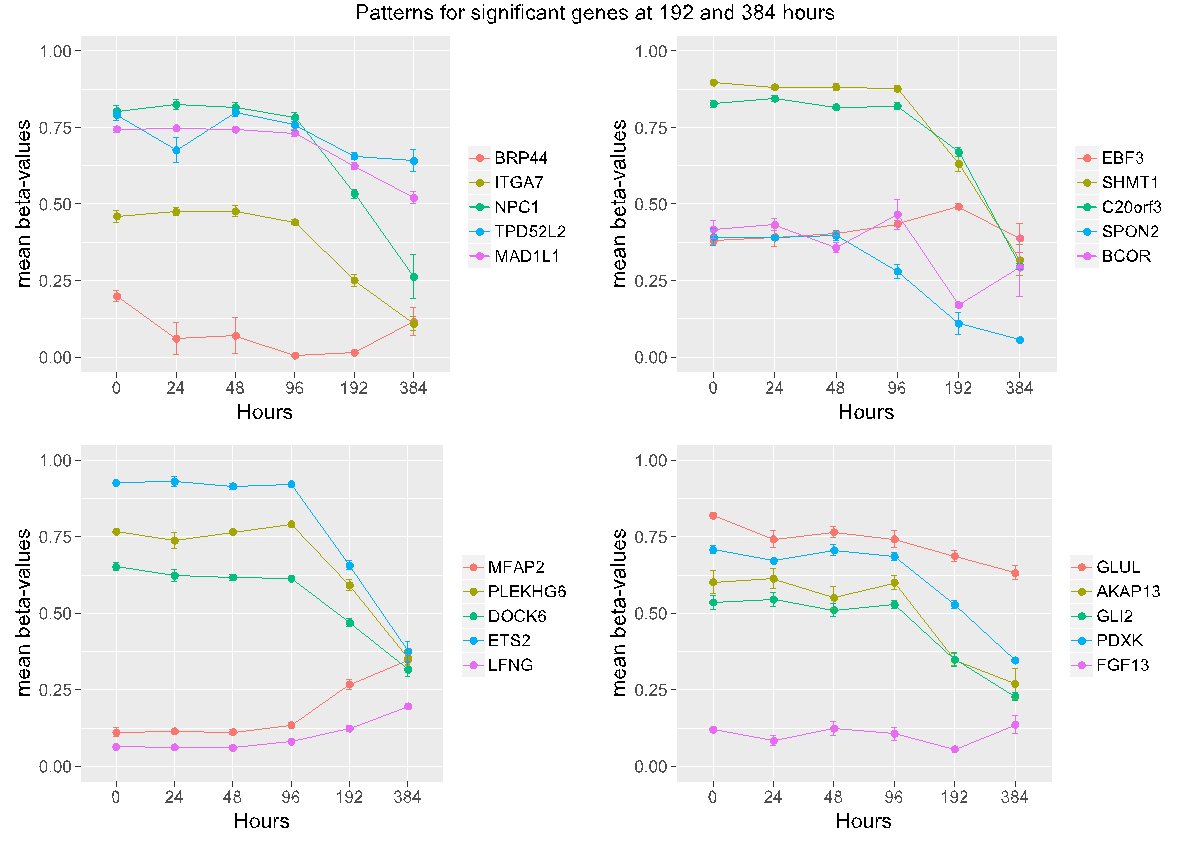


Supplementary Figure S1: Methylation patterns during differentiation for the 20 genes showing significant methylation changes both at 192 and 384 hours. To better inspect the patterns, genes are separated in four panels. Dots define averaged β-values for each time-point, while different colors represent different genes.


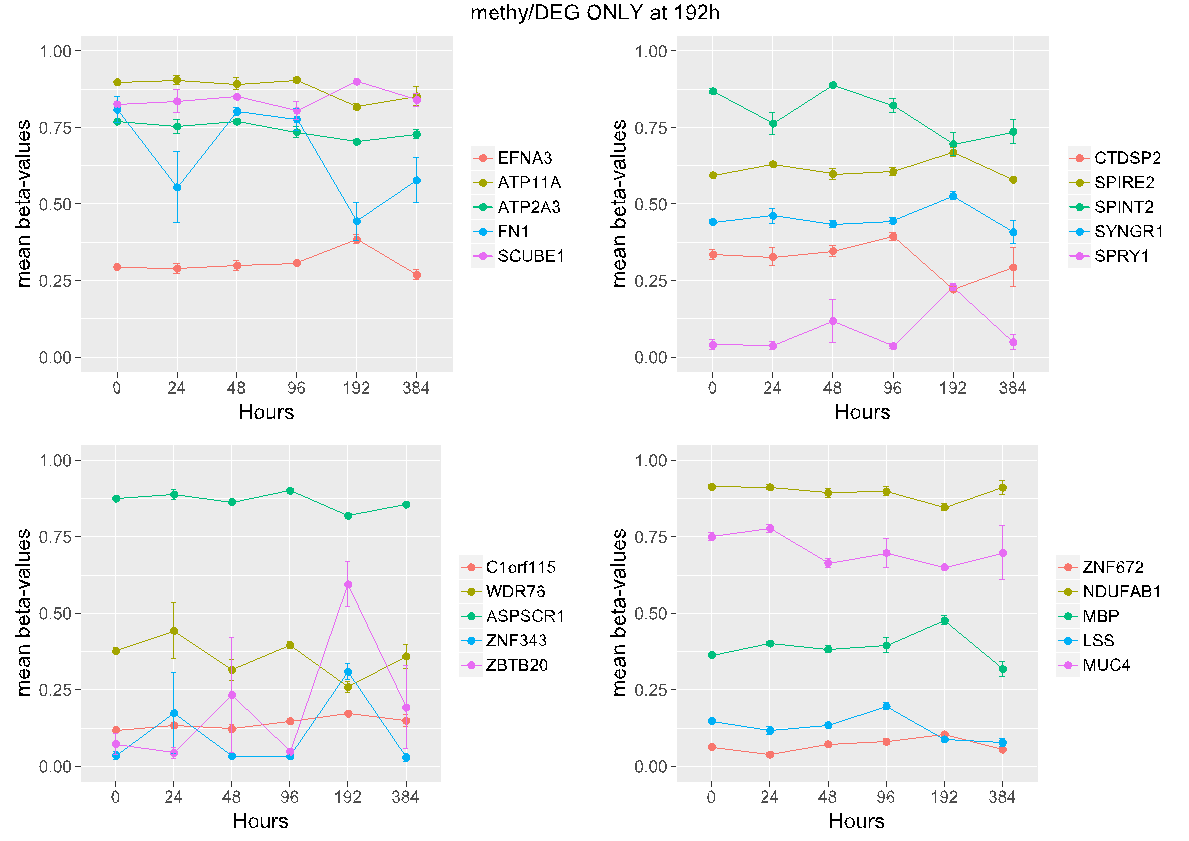


Supplementary Figure S2: methylation patterns during differentiation for the 20 genes significantly methylated/expressed at 192 hours (but not at 384 hours) which methylation levels return to the baseline at 384 hours. For each panel, mean β-values at each time point are represented by dots. Different colors represent different genes.


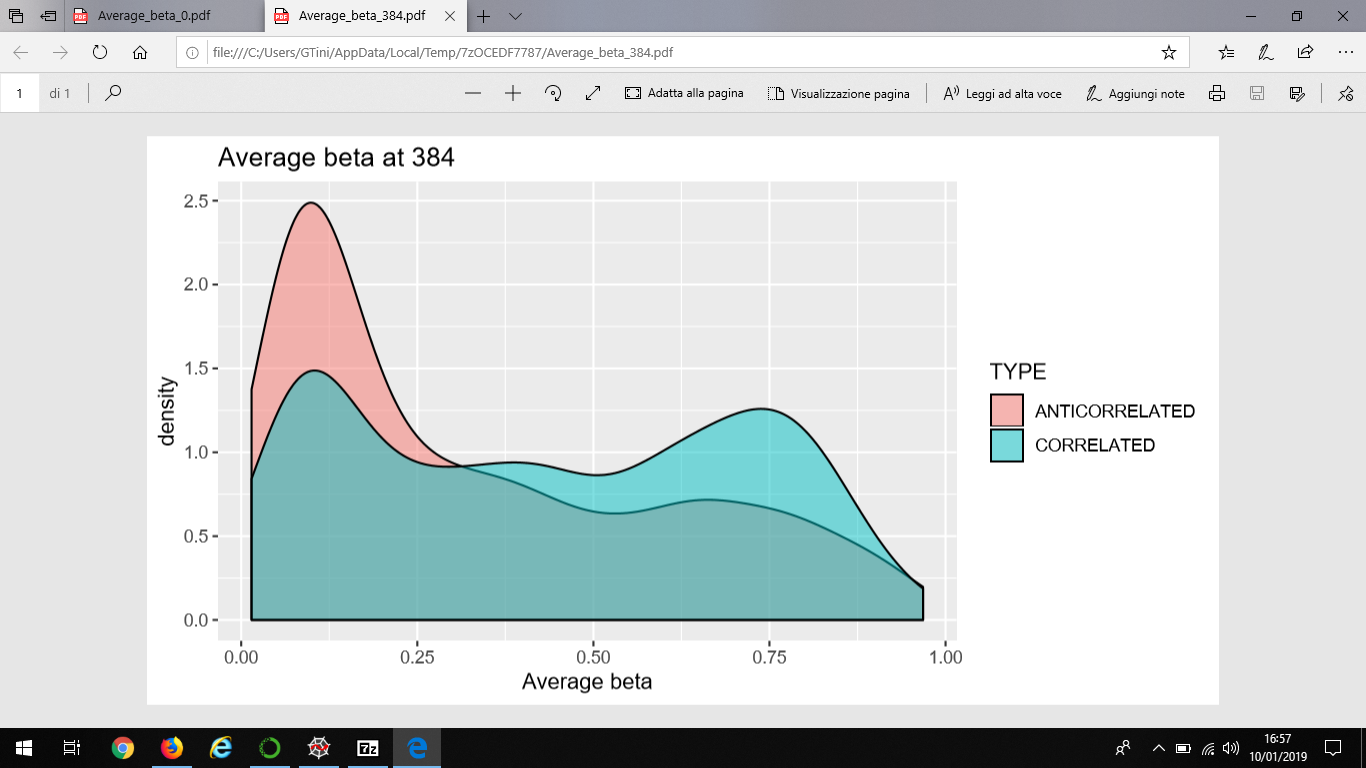


Supplementary Figure S3: Distribution of the average beta-values for the DMRs on DEGs at 384 hours. β-values of DMRs that show anti-correlated changes in methylation and gene expression are depicted in pink. The blue curve describes instead the distribution of β-values for DMRs showing the same direction of regulation in methylation and gene expression levels.
